# Risk of post-acute symptoms among adults: A comparison study of severe COVID-19, pneumonia, and influenza

**DOI:** 10.1371/journal.pone.0322020

**Published:** 2025-04-29

**Authors:** Caroline M. Schaefer, Trudy Millard Krause, George L. Delclos, Raymond S. Greenberg

**Affiliations:** 1 Department of Management, Policy and Community Health, School of Public Health, University of Texas Health Science Center at Houston, Houston, Texas United States of America; 2 Southwest Center for Occupational and Environmental Health, School of Public Health, University of Texas Health Science Center at Houston, Houston, Texas United States of America; 3 Peter O’Donnell Jr. School of Public Health, University of Texas Southwestern Medical Center, Dallas, Texas United States of America; Carol Davila University of Medicine and Pharmacy, ROMANIA

## Abstract

**Background:**

A retrospective cohort study was undertaken to assess the relationship between initial disease severity of COVID-19 and the risk of post-acute symptoms. The COVID-19 cohort was compared against influenza and pneumonia cohorts to assess whether risk of post-acute symptoms differed.

**Methods:**

Administrative health claims data were obtained for commercially insured and Medicare Advantage covered adults (*≥* 18 years) with symptomatic laboratory-confirmed COVID-19 diagnosed in 2020 (n=121,205), and similar cohorts of influenza (n=20,844) and pneumonia (n=29,052) patients diagnosed prior to the pandemic. Post-acute symptoms were assessed at four weeks, three and six months following initial diagnosis.

**Results:**

Among the patients with COVID-19, the likelihood of any post-acute symptom increased with initial disease severity, and was also influenced by the presence of comorbidities, especially rheumatoid arthritis, ischemic heart disease and asthma. The specific post-acute symptoms varied by age, with increased risks of anxiety and headache among the young, whereas the elderly experienced increased brain fog and fatigue. When compared against the influenza and pneumonia cohorts, all three groups experienced post-acute symptoms, with a strong relationship to disease severity, and only partial resolution over the six-month observation period. Those with influenza were less likely than those with COVID-19 to experience post-acute symptoms while those with pneumonia were more likely to have post-acute illness than those with COVID-19.

**Conclusions:**

Using a large national dataset, we found that COVID-19 symptomology could not be described by previously seen influenza or pneumonia symptomology and differences exist in the prevalence of symptoms as well as time to resolution, better characterizing “long COVID” and identifying that these differences are unique to COVID-19.

## Background

According to the American Journal of Public Health, approximately 3.58 million COVID-19 hospitalizations occurred from May 2020 through April 2021 in the United States [1]. About 80% of patients with coronavirus disease 2019 (COVID-19) experienced mild or no symptoms [2]. Among symptomatic COVID-19 patients, about two-thirds return to their usual state-of-health within one to two months of diagnosis, whereas others experience prolonged recoveries marked by a diverse set of symptoms, most commonly including fatigue, join pain, dyspnea, or memory problems [2].

The US Centers for Disease Control and Prevention (CDC) defines long COVID as marked by sequelae that extend at least three months after initial infection [3]. However, other studies identified the diagnosis of post-acute COVID-19 as symptoms continuing up to 120 days beyond recovery [4]. The systematic review by Chen, et. al. found post COVID-19 symptom pooled prevalence among patients to be 32% (95%CI: [14%, 57%]) after 90 days and 49% (95%CI: [40%, 59%]). However many of the studies in the symptomatic review which measured symptomology that far out after initial infection concentrated on hospitalized populations, highlighting the need for measurement among more generalizable populations.

Several studies have shown that, while more likely among the most severe initial COVID-19 infection, the symptoms of long COVID can present among those with very mild initial acute disease as well. A retrospective nationwide cohort study in Israel including 1.9 million subjects showed that even mild COVID-19 infections led to increased risk of anosmia (loss of smell) and dysgeusia (abnormal taste), concentration and memory difficulties, dyspnea, and weakness up to 180–360 days post-infection [5]. It has also been shown that COVID-19 patients who required either non-critical or critical hospital admissions were more likely to experience post-acute COVID-19 symptoms after 90 days than those who could be treated via outpatient services [6]. A 2021 Swedish study of more than 2,000 subjects revealed two-to-three-month recoveries among those with self-reported severe infections versus up to two-week recovery for mild infections [7].

Some previous studies have noted differences in long-term sequelae of COVID-19 as compared to those occurring after acute influenza. A study looking at patients hospitalized for COVID-19 versus seasonal influenza found that COVID-19 hospitalization was associated with higher long-term risk of adverse outcomes in nine organ systems (cardiovascular, coagulation and hematological, fatigue, gastrointestinal, kidney, mental health, metabolic, musculoskeletal, neurological) but a lower risk of pulmonary outcomes [8]. A cohort study of Medicare beneficiaries compared long COVID patients and influenza patients who met the same symptom definitions as long COVID and defined it as “long influenza” [9]. They found that rates of long symptomology were similar for influenza and COVID outpatients (17.0% and 16.6%) and inpatients (24.6% and 29.2%). However, the symptomology between the two differed. Long COVID patients had higher incidences of dyspnea, fatigue, loss of taste and smell, and neurocognitive symptoms compared to long flu.

Although post-acute COVID-19 has been studied repeatedly, few studies focused on more nuanced delineations of severity of the acute illness or compared the phenomenon to the sequelae of other acute major infectious respiratory disease. The primary objective of our study was to examine post-acute COVID-19 risk across severity levels of symptomatic COVID-19 disease among adults in a large U.S. national administrative claims dataset. This source of data offered the advantage of tracking patients across any treating provider. Separate analyses focused on individual and overall post-acute symptoms and compared the findings between those with pre-existing comorbid conditions to those without disease history. Secondary goals included examination of the time course to resolution of post-acute symptoms and to assess whether patients with COVID-19 experience more frequent post-acute symptoms than patients with other common pulmonary infections. Influenza and pneumonia were selected for comparison purposes because of their respiratory effects and possible treatment with mechanical ventilation and intensive care.

## Methods

### Data source

The study data were extracted from Optum’s Clinformatics® Data Mart (CDM) (OptumInsight, Eden Prairie, MN) which is derived from administrative health claims for members of large commercial and Medicare Advantage health plans on April 5, 2022. The database includes all 50 states, with more than 17 million covered lives annually, for a total of over 65 million unique lives over a nine-year period from January 2007 through December 2020 [10]. Information was de-identified concerning protected health information under the Expert Determination method consistent with HIPAA [11] and managed according to Optum® customer data use agreements. CDM administrative claims submitted for payment for all medical and pharmacy health care services are verified, adjudicated and de-identified prior to inclusion. As the COVID-19 pandemic began in the United States in early 2020 and continues to date, claims through September 30, 2021 were included for data extraction in April 2022.

This study was reviewed and approved by the Committee for the Protection of Human Subjects of the University of Texas Health Science Center at Houston. This report adheres to the STROBE (Strengthening the Reporting of Observational Studies in Epidemiology) [12] statement guidelines.

### Cohort selection

A total of 132,332 subjects were eligible for the study because they met the following inclusion criteria: had a lab-confirmed or clinically diagnosed COVID-19 (ICD-10-CM I07.1) diagnosis in 2020, had continuous inclusion in the administrative claim database from January through December of 2020, were 18 years of age or older at the time of diagnosis, and reported symptoms at the time of diagnosis. After removing subjects with missing demographic information (8.4%), the final COVID-19 cohort with complete records was 121,205 individuals. The same criteria were used for an influenza population during the 2018–2019 influenza season, October 2018 to March 2019, resulting in a cohort of 20,844 (ICD-10-CM J09.x, J10.x, J11.x). An additional pneumonia cohort was created using subjects diagnosed with pneumonia between January and December of 2018, culminating in a pneumonia cohort with 29,052 subjects (ICD-10-CM J12.x-J18.x). The influenza and pneumonia cohorts were assembled prior to the COVID-19 pandemic to eliminate the possibility that symptoms after disease diagnosis were caused by a subsequent COVID-19 illness.

### Study variables

The primary dependent variable (outcome) of interest in this investigation was the presence (= 1) or absence (= 0) of symptoms at three timepoints of interest after a diagnosis of COVID-19: 4 weeks, 3 months, and 6 months. The individual types of symptoms considered were those identified in prior research: anosmia and/or ageusia, anxiety, arrythmias, brain fog, chest pain, cough, dyspnea, fatigue, general pain, headache, insomnia, joint pain, and muscle weakness [13]. Other potential sequelae, such as post-traumatic stress disorder, persistent supplemental oxygen requirement, hair loss, thromboembolic events, and dysautonomia, were reported in 0.1% or fewer COVID-19 subjects, and therefore, were not included in the analysis.

Potential predictors of persistent symptoms included the following demographic characteristics: (gender, 0 = male, 1 = female), race/ethnicity coded in four binary dummy variables (Non-Hispanic White, Non-Hispanic Black, Hispanic, Asian), age coded in seven ordinal categories (18–29, 30–39, 40–49, 50–59, 60–69, 70–79, 80+ years), and U.S. region (South, Midwest, Northeast, West) coded in four binary dummy variables. Clinical predictors included the following comorbidities: diabetes, hypertension, obesity, ischemic heart disease, chronic renal disease, chronic obstructive pulmonary disease, asthma, rheumatoid arthritis, and dementia (each coded as absent = 0, present = 1). In addition, an index of disease severity was created as an adaptation of one proposed originally by the World Health Organization (WHO) [14]. To distinguish the most severe levels of COVID-19 illness, the WHO ten-point scale requires information about oxygen levels during mechanical ventilation that are not routinely available in administrative claims data. As an approximation to the WHO index, we created a nine-level scale (1 = self-report of diagnosed asymptomatic disease; 2 = confirmed asymptomatic disease; 3 = symptomatic but ambulatory; 4 = symptomatic and visited an emergency department but was not admitted; 5 = admitted to a hospital but did not require supplemental oxygen; 6 = admitted to a hospital and received non-invasive supplemental oxygen, including via high-flow nasal cannula; 7 = admitted to a hospital and required mechanical ventilation; 8 = admitted to a hospital and received mechanical ventilation plus either renal dialysis or ECMO; 9 = deceased). This severity scale was applied to all three disease cohorts. For the purposes of the present analysis, we excluded the subjects with self-reported infections (severity level 1, because of inability to confirm diagnosis), those who were asymptomatic at time of diagnosis (severity level 2, because persistence of symptoms was not relevant) and those who died (severity level 9, because of lack of a post-acute phase), leaving six ordinal levels of disease severity (3–8).

### Statistical analysis

The distributions of independent variables and prevalence of post-acute symptoms were examined comparatively between the three disease cohorts. Statistical significance (alpha = 0.05) for differences in the distributions was assessed by the chi square test. Logistic regression was used to evaluate the relationships between post-acute symptoms and infectious agent while adjusting for independent variables. Additional models were developed to assess the relationship between COVID-19 severity and post-acute symptoms among only the COVID-19 cohort. The fully adjusted models included all the demographic, comorbidity, and severity variables. Odds ratios and 95% confidence intervals (CIs) were presented as adjusted measures of association between post-acute symptoms and each of the independent variables.

## Results

Demographic characteristics, comorbidities, and illness severity of the three cohorts are shown in Table 1. While all variables showed statistically significant differences between the three cohorts (p < 0.001), there were common trends for some. For all cohorts, the most common race was Non-Hispanic White, gender was female, and United States region was the South. This follows the underlying demographic distribution of the Optum Clinformatics® Data Mart database. However, differing trends between cohorts were seen among age groups, comorbidities, and illness severity. Within the COVID-19 cohort, the distribution of age was relatively uniform across age groups, while the influenza cohort age distribution trended towards younger groups and pneumonia was skewed towards older groups. For all cohorts, hypertension was the most common comorbidity. The second most common comorbidity was obesity in COVID-19 and influenza cohorts, but for those with pneumonia, it was COPD. Overall, the pneumonia cohort had higher frequencies of all comorbidities except for obesity, and influenza the lowest frequencies except for asthma.

**Table 1:** Characteristics of disease cohorts.

	Disease Cohort						p value
	COVID-19		Influenza		Pneumonia		
All	121,205	--	20,844	--	29,052	--	
Age Group (N, %)							< 0.001
18–29	13,625	11.24%	3,490	16.74%	1,108	3.81%	
30–39	12,003	9.90%	4,017	19.27%	1,790	6.16%	
40–49	15,668	12.93%	3,891	18.67%	2,465	8.48%	
50–59	19,422	16.02%	3,617	17.35%	4,068	14.00%	
60–69	20,415	16.84%	2,627	12.60%	5,082	17.49%	
70–79	23,781	19.62%	1,927	9.24%	6,413	22.07%	
80+	16,291	13.44%	1,275	6.12%	8,126	27.97%	
Gender (N, %)							< 0.001
Female	64,507	53.22%	11,875	56.97%	15,992	55.05%	
Male	56,698	46.78%	8,969	43.03%	13,060	44.95%	
Race (N, %)							< 0.001
Asian	3,369	2.78%	768	3.68%	652	2.24%	
Black	16,210	13.37%	2,557	12.27%	3,647	12.55%	
Hispanic	24,913	20.55%	3,196	15.33%	2,274	7.83%	
White	76,713	63.29%	14,323	68.72%	22,479	77.38%	
Region (N, %)							< 0.001
Midwest	30,979	25.56%	4,506	21.62%	8,782	30.23%	
Northeast	10,429	8.60%	1,997	9.58%	3,672	12.64%	
South	59,918	49.44%	11,823	56.72%	12,798	44.05%	
West	19,879	16.40%	2,518	12.08%	3,800	13.08%	
Comorbidities (N, %)							
Diabetes	39,082	32.24%	3,751	18.00%	10,172	35.01%	< 0.001
Asthma	17,683	14.59%	3,249	15.59%	6,441	22.17%	< 0.001
Hypertension	72,637	59.93%	8,823	42.33%	21,729	74.79%	< 0.001
Ischemic Heart Disease	24,518	20.23%	2,518	12.08%	10,272	35.36%	< 0.001
COPD	25,205	20.80%	4,278	20.52%	14,091	48.50%	< 0.001
Rheumatoid Arthritis	9,850	8.13%	1,511	7.25%	3,444	11.85%	< 0.001
Dementia	11,432	9.43%	572	2.74%	3,638	12.52%	< 0.001
Obesity	41,141	33.94%	5,255	25.21%	8,657	29.80%	< 0.001
Severity (N, %)							< 0.001
3 – Symptomatic	47,060	38.83%	13,621	65.35%	10,018	34.48%	
4 - ED Visit	37,525	30.96%	5,385	25.83%	5,425	18.67%	
5 - Hospitalization, no higher treatment	14,594	12.04%	343	1.65%	2,436	8.38%	
6 - Hosp w/ non-invasive oxygen	8,594	7.09%	278	1.33%	2,054	7.07%	
7 - Hosp w/ mechanical vent	12,550	10.35%	1,166	5.59%	8,673	29.85%	
8 - Hosp w/ mechanical vent and Renal Dialysis or ECMO	882	0.73%	51	0.24%	446	1.54%	

The most common post-acute symptoms (Table 2) after 4 weeks among the COVID-19 cohort were fatigue (25.4%), dyspnea (24.7%), and joint pain (24.7%). After 4 weeks in the influenza cohort, the three most common symptoms were joint pain (19.8%), fatigue (18.2%), and cough (17.7%). For the pneumonia cohort, dyspnea (42.2%), fatigue (34.8%), and cough (33.9%) were the three most common after 4 weeks.

**Table 2:** Presence of symptoms by cohort over time.

		4 weeks		3 months		6 months		†P value
Illness Cohort	Symptom	N	%	N	%	N	%	
COVID-19	Anxiety	17,216	14.20%	14,600	12.05%	11,287	9.31%	< 0.001
	Arrythmias	10,600	8.75%	8,630	7.12%	6,229	5.14%	< 0.001
	Brain Fog	1,705	1.41%	1,386	1.14%	1,037	0.86%	< 0.001
	Chest Pain	14,977	12.36%	11,971	9.88%	8,384	6.92%	< 0.001
	Cough	21,349	17.61%	16,407	13.54%	11,967	9.87%	< 0.001
	**Dyspnea**	**29,923**	**24.69%**	**23,763**	**19.61%**	**17,254**	**14.24%**	< 0.001
	**Fatigue**	**30,793**	**25.41%**	**25,070**	**20.68%**	**18,460**	**15.23%**	< 0.001
	Headache	11,022	9.09%	9,663	7.97%	7,091	5.85%	< 0.001
	**Joint Pain**	**29,876**	**24.65%**	**26,309**	**21.71%**	**20,148**	**16.62%**	< 0.001
Influenza	Anxiety	2,387	11.45%	2,063	9.90%	1,476	7.08%	< 0.001
	Arrythmias	1,235	5.92%	1,040	4.99%	731	3.51%	< 0.001
	Brain Fog	118	0.57%	95	0.46%	66	0.32%	< 0.001
	Chest Pain	1,898	9.11%	1,558	7.47%	1,019	4.89%	< 0.001
	**Cough**	**3,674**	**17.63%**	**2,906**	**13.94%**	**2,110**	**10.12%**	< 0.001
	Dyspnea	3,169	15.20%	2,589	12.42%	1,829	8.77%	< 0.001
	**Fatigue**	**3,770**	**18.09%**	**3,154**	**15.13%**	**2,178**	**10.45%**	< 0.001
	Headache	422	2.02%	352	1.69%	244	1.17%	< 0.001
	**Joint Pain**	**4,123**	**19.78%**	**3,546**	**17.01%**	**2,543**	**12.20%**	< 0.001
Pneumonia	Anxiety	5,386	18.54%	4,631	15.94%	3,652	12.57%	< 0.001
	Arrythmias	3,711	12.77%	3,052	10.51%	2,259	7.78%	< 0.001
	Brain Fog	569	1.96%	481	1.66%	344	1.18%	< 0.001
	Chest Pain	5,847	20.13%	4,766	16.41%	3,452	11.88%	< 0.001
	**Cough**	**9,859**	**33.94%**	**8,032**	**27.65%**	**5,959**	**20.51%**	< 0.001
	**Dyspnea**	**12,225**	**42.08%**	**10,315**	**35.51%**	**8,054**	**27.72%**	< 0.001
	**Fatigue**	**10,123**	**34.84%**	**8,422**	**28.99%**	**6,385**	**21.98%**	< 0.001
	Headache	600	2.07%	510	1.76%	356	1.23%	< 0.001
	Joint Pain	8,808	30.32%	7,675	26.42%	5,885	20.26%	< 0.001

† p-value resulting from Chi-Square test of proportions of the symptom in the three different time periods

### COVID-19 cohort

**Fig 1:**
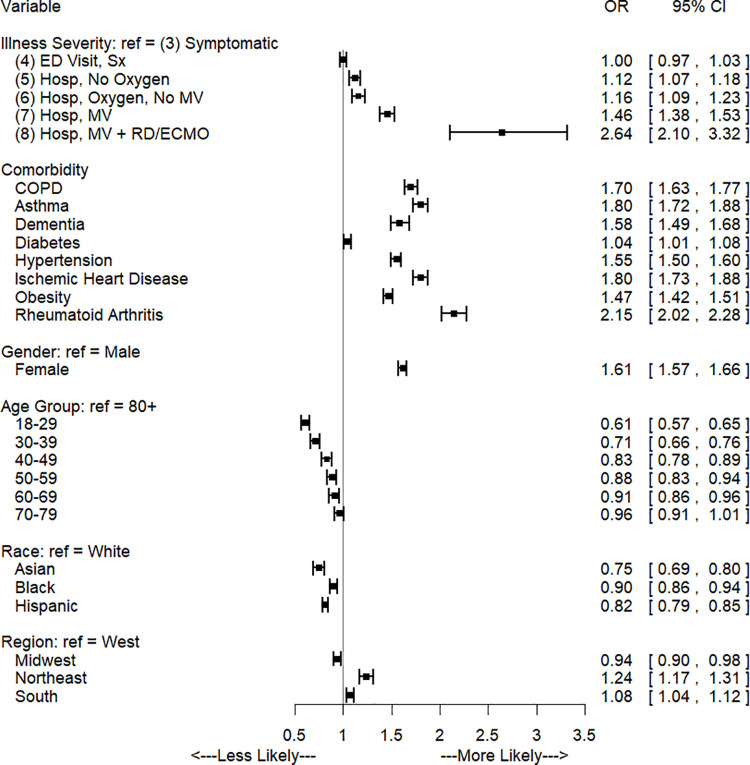
Odds of any symptom 4 weeks after initial diagnosis, COVID-19 cohort only.

**Fig 2:**
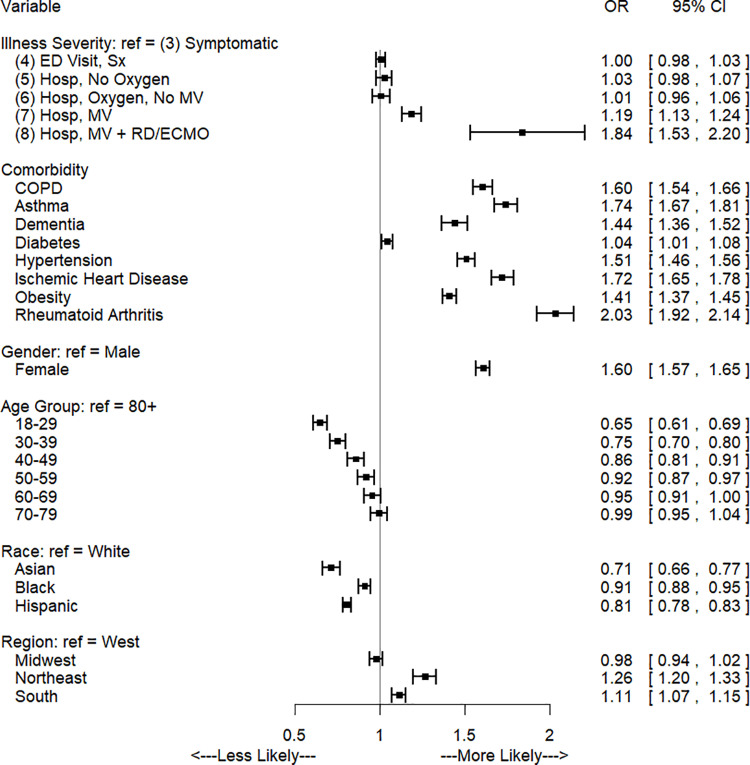
Odds of any symptom 3 months after Initial Diagnosis, COVID-19 cohort only.

**Fig 3:**
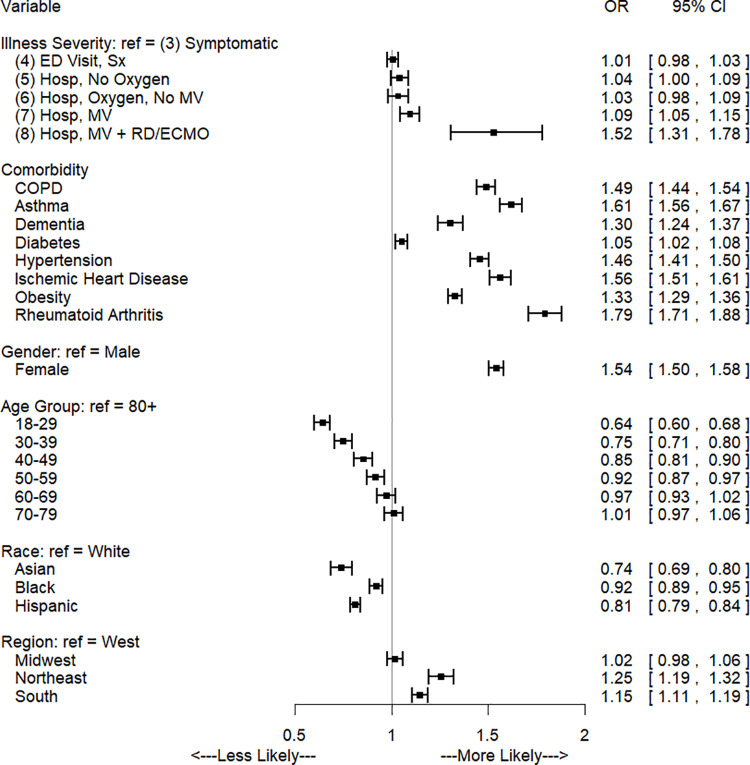
Odds of any symptom 6 months after initial diagnosis, COVID-19 cohort only.

Odds ratios and confidence intervals for the regression modeling symptoms after 4 weeks for the COVID-19 cohort are shown in Fig 1. Odds of any symptom were not significantly different between illness severity levels 4 and 3. However after 4 weeks, odds of any symptom for severity levels 5, 6, 7, and 8 were 1.12 [95% CI: 1.07. 1.18], 1.16 [1.09, 1.23], 1.46 [1.38, 1.53], and 2.64 [2.10, 3.32] times the odds for severity level 3, respectively. As post-diagnosis time progressed, only the most severe cases continued to present symptoms (severity levels 7 and 8. For level 7, the odds ratio of having a symptom versus severity level 3 was 1.19 [1.13, 1.24] and 1.10 [1.05, 1.15] after 3 and 6 months. Similarly, level 8 odds ratio was 1.84 [1.53, 2.21] and 1.53 [1.31, 1.78] after 3 and 6 months. Presence of a comorbidity resulted in an increased odds for all time points and comorbidities. The comorbidities with the largest increased odds of symptoms after 4 weeks were rheumatoid arthritis (OR: 2.15 [2.02, 2.28]), ischemic heart disease (OR: 1.80 [1.73, 1.88]), and asthma (OR: 1.80 [1.72, 1.88]). Overall, compared with those aged 80 and older, odds of any symptom after 4 weeks were lower for all age groups except for age 70–79, which was not statistically different. Figs 2 and 3 depict odds ratios for 3 and 6 months post-COVID and a full listing of all odds ratios at each time point are in supplementary tables in the appendix.

Odds of a diagnosis of anxiety, brain fog, fatigue, and dyspnea, after 4 weeks, 3 months, and 6 months increased as COVID-19 severity level increased when controlling for demographics and comorbidities. Odds of fatigue after 4 weeks for those with severity level 8 was 3.32 [2.88, 3.82] times level 3 odds, and odds ratio of dyspnea after 4 weeks was 3.07 [2.65, 3.54]. As time progressed, the magnitude of odds ratios decreased but maintained the trend. For arrythmias, chest pain, and headache, severity levels 4 and 8 had consistently higher odds of the specified symptom than severity level 3. Odds ratio of arrythmia for severity level 8 versus level 3 was 1.74 [1.46, 2.07] after 4 weeks, 1.63 [1.35, 1.97] after 3 months, and 1.51 [1.22, 1.88] after 6 months. Odds ratio of chest pain for severity level 8 versus level 3 was 1.64 [1.40, 1.91] after 4 weeks, 1.57 [1.33, 1.86] after 3 months, and 1.53 [1.27, 1.84] 6 months. Odds ratio of headache for severity level 8 versus level 3 was 1.57 [1.30, 1.90] after 4 weeks, 1.61 [1.32, 1.97] after 3 months, and 1.64 [1.30, 2.05] after 6 months. Odds of cough was less likely among most severity levels than severity level 3 except for level 8, although the difference did not reach statistical significance. There were no statistically significant differences between severity levels for odds of joint pain except for severity level 5, which had an odds ratio of 0.94 [0.89, 0.98] after 3 months and 0.94 [0.90, 0.995] after 6 months after COVID-19 diagnosis.

Anxiety and headache were more likely among all age groups compared to those aged 80 and over. The odds ratio of anxiety for those age 30–39 versus individuals 80 and over 2.34 [2.15, 2.55] after 4 weeks, 2.38 [2.17. 2.61] after 3 months, and 2.35 [2.12, 2.60] after 6 months among those aged 30–39 compared to those 80 and older. Odds ratio of headache was highest among 18- to 29-year-olds after 4 weeks and 3 months (2.43 [2.19, 2.70] and 2.42 [2.17, 2.71] times the odds in those 80 and older, respectively) and highest for aged 30–39 after 6 months (2.43 [2.14, 2.76]). Brain fog and fatigue were less likely among all age groups compared to those aged 80 and older. Odds of brain fog were lowest among 18- to 29-year-olds after 4 weeks, 3 months, and 6 months (0.06 [0.03, 0.12], 0.05 [0.03, 0.11], and 0.04 [0.02, 0.10] times the odds those aged 80 and older). Similarly, odds of fatigue were lower among 18–29-year-olds after all timepoints (4 weeks: 0.43 [0.40, 0.46], 3 months: 0.45 [0.42, 0.49], 6 months: 0.43 [0.39, 0.48]). Odds ratio of joint pain after 4 weeks for ages 18–29, 30–39, and 40–49 were 0.45 [0.42, 0.49], 0.53 [0.49, 0.57], and 0.77 [0.72, 0.82] times the odds in those 80 and older, respectively. Similar odds ratios were seen after 3 months and 6 months. Odds ratio of dyspnea after 4 weeks for ages 18–29 and 30–39 are 0.58 [0.54, 0.63] and 0.80 [0.74, 0.86] times the odds for those 80 and older. 18–29-year-olds had the lowest odds of arrythmias after all time points.

Having the co-morbid conditions of COPD or asthma with COVID-19 increased the odds of all symptoms at all time points. The largest increase in odds after 4 weeks was for dyspnea (COPD: 1.98 [1.92, 2.05], asthma: 1.83 [1.76, 1.90]) and cough (COPD: 1.77 [1.70, 1.83], asthma: 1.76 [1.69, 1.83]). After 4 weeks, having dementia increased the odds of anxiety (2.42 [2.89, 2.56]), brain fog (3.39 3.822]), cough (1.30 [1.23, 1.97]), and fatigue (1.77 [1.69, 1.85]). Having hypertension increased the odds of all symptoms with the largest increases being chest pain 1.72 [1.63, 1.82]) and arrythmias (1.68 [1.58, 1.79]) after 4 weeks. Also increasing the odds of all symptoms was ischemic heart disease, which had the largest increase for chest pain (2.22 [2.13, 2.32]) and arrythmias (2.13 [2.03, 2.24]). Being obese increased the odds of all symptoms. The symptoms with the largest increase in odds due to rheumatoid arthritis were joint pain (2.12 [2.03, 2.22]), headache (1.55 [1.46, 1.65]), and fatigue (1.52 [1.45, 1.59]).

The Northeast region of the United States had the highest rates of all post-acute symptoms in the COVID-19 cohort, except for headache, for which the Northeast had the lowest odds, and brain fog and joint pain, which were not statistically different from the West region. White individuals were the most likely to have anxiety or fatigue compared to Asian, Black, and Hispanic individuals. Hispanic individuals were least likely to have post-acute arrythmias, brain fog, dyspnea.

### Comparison of COVID-19 cohort to influenza and pneumonia cohorts

The overall prevalence of symptoms at 4 weeks, 3 months, and 6 months after diagnosis by severity level for each disease cohort is shown in Table 3. After 4 weeks, 3 months, and 6 months of diagnosis, the odds ratios of any symptom in the influenza cohort versus the COVID-19 cohort were 0.77 [0.75, 0.80], 0.77 [0.75, 0.80], and 0.68 [0.66, 0.71], respectively. Conversely, the odds ratios in the pneumonia cohort were 2.24 [2.16, 2.32], 2.41 [2.33, 2.49], and 2.46 [2.39, 2.54].

**Table 3:** Presence of post-acute symptoms in relation to illness severity among disease cohorts.

		Time from Diagnosis						
		After 4 weeks		After 3 months		After 6 months		†P value
Disease Cohort	Severity	N	%	N	%	N	%	
COVID-19	3 - Symptomatic	27,971	59.44%	25,350	53.87%	20,889	44.39%	< 0.001
	4 - ED Visit	22,798	60.75%	20,700	55.16%	17,123	45.63%	< 0.001
	5 - Hospitalization, no oxygen therapy	11,129	76.26%	10,046	68.84%	8,585	58.83%	< 0.001
	6 - Hosp w/ non-invasive oxygen	6,494	75.56%	5,788	67.35%	4,958	57.69%	< 0.001
	7 - Hosp w/ mechanical vent	10,188	81.18%	9,154	72.94%	7,739	61.67%	< 0.001
	8 - Hosp w/ mechanical vent and Renal Dialysis or ECMO	795	90.14%	729	82.65%	629	71.32%	< 0.001
Influenza	3 - Symptomatic	6,843	50.24%	6,081	44.64%	4,737	34.78%	< 0.001
	4 - ED Visit	2,951	54.80%	2,668	49.55%	2,115	39.28%	< 0.001
	5 - Hospitalization, no higher treatment	247	72.01%	225	65.60%	173	50.44%	< 0.001
	6 - Hosp w/ non-invasive oxygen	216	77.70%	195	70.14%	155	55.76%	< 0.001
	7 - Hosp w/ mechanical vent	961	82.42%	881	75.56%	735	63.04%	< 0.001
	8 - Hosp w/ mechanical vent and Renal Dialysis or ECMO	45	88.24%	42	82.35%	38	74.51%	0.198
Pneumonia	3 - Symptomatic	7,178	71.65%	6,531	65.19%	5,553	55.43%	< 0.001
	4 - ED Visit	3,898	71.85%	3,588	66.14%	3,084	56.85%	< 0.001
	5 - Hospitalization, no higher treatment	2,044	83.91%	1,876	77.01%	1,650	67.73%	< 0.001
	6 - Hosp w/ non-invasive oxygen	1,736	84.52%	1,596	77.70%	1,378	67.09%	< 0.001
	7 - Hosp w/ mechanical vent	7,576	87.35%	6,998	80.69%	6,195	71.43%	< 0.001
	8 - Hosp w/ mechanical vent and Renal Dialysis or ECMO	425	95.29%	394	88.34%	354	79.37%	< 0.001

† p-value resulting from Chi-Square test of proportions of a symptom in the three different time periods

The most frequently reported specific symptom in the COVID-19 cohort was fatigue (25.4% at 4 weeks), joint pain for the flu cohort (19.8% at 4 weeks), and shortness of breath in the pneumonia cohort (42.1% at 4 weeks). After six months, the symptoms with the greatest prevalence stayed the same for the influenza and pneumonia cohorts but was instead joint pain for the COVID-19 cohort. Specific symptom prevalence over time for all three cohorts are shown in Table 2. Generally, for the specific symptoms, odds of a symptom was lower for the influenza cohort than the COVID-19 cohort except for cough, for which there was no statistical difference. This was true at all three timepoints of interest. The greatest difference in symptom odds was for headache, for which the influenza cohort had 0.18 [0.16, 0.19] times the odds after 4 weeks than COVID-19. A similarly consistent trend was seen among the pneumonia cohort having a higher odds of all of the specific symptoms except for brain fog and headache when compared to COVID-19. Cough had the largest odds ratio for pneumonia compared to COVID-19 after 4 weeks (2.59 [2.51, 2.67]. Odds of headache was the only symptom for which pneumonia had a lower odds than COVID-19, 0.27 [0.25, 0.29] times the odds after 4 weeks. Odds of brain fog was not shown to be different between the pneumonia and COVID-19 cohorts. These were true at all three time points as well.

## Discussion

It is well-recognized that acute COVID-19 infection may be followed by persistent symptoms. Our findings support prior observations that the most common persistent symptoms are fatigue, joint pain and dyspnea. The literature on the effect of comorbidities has been inconsistent. A 2023 meta-analysis of 41 studies that investigated risk factors or predictors of post-COIVD conditions found that. Using pooled risk estimates, anxiety or depression, asthma, COPD, diabetes, immunosuppression, and ischemic heart disease all increased risk of these conditions [15]. However, not all of the individual studies came to the same conclusions. In the study Oelsner et. al., obesity, diabetes, asthma, COPD, and elevated depressive symptoms were not associated with post-COVID recovery [6]. It is not surprising that a variety of conditions that predispose to severe acute COVID-19 also are linked to post-acute manifestations. Our examination of comorbidities found the strongest risk of post-acute COVID-19 syndrome among those with rheumatoid arthritis, ischemic heart disease, and asthma. Since chest pain, joint pain and dyspnea are common post-acute sequelae, conditions capable of producing those symptoms might amplify risk.

We observed that development of post-acute symptoms was more likely to arise among females. This has been reported previously in a variety of settings [6,15,16], so it appears to be a reproducible finding. During the SARS outbreak of 2003, it was observed that women survivors manifested higher levels of stress, depression and anxiety following a SARS infection [17].

Although racial and ethnic minorities, particularly non-Hispanic Blacks and Hispanics, are at increased risk of acute COVID-19, [17] our investigation found mild reductions in post-acute adjusted risks among non-Hispanic Blacks and Hispanics. Since our study population included only persons with health insurance, thereby limiting known racial/ethnic disparities in access to care in the United States, [18] the observed small racial/ethnic post-acute risk may underestimate differences in the general population.

An unexpected finding was a regional difference in post-acute COVID-19, with patients from the Northeast manifesting the greatest risk. This phenomenon was observed for any symptoms as well as for each of the most common individual symptoms. The consistency of these findings suggests that there was a systematically higher risk in the Northeast. We are not aware of any prior report of regional differences in the occurrence of post-acute sequelae. One possible explanation is that the Northeast was impacted disproportionately by the initial wave of COVID-19, before effective treatments and management guidelines were established [18]. It seems plausible that care disparities may account, at least partially, for the observed elevated post-acute risk. Also, social circumstances in the highly urbanized Northeast may be partly responsible. A study conducted in New York, New Jersey and Connecticut revealed COVID-19 was associated with overcrowding, income inequality and lack of health insurance [19].

A notable finding in this study was the powerful relationship between severity of COVID-19 and persistent symptoms beyond four weeks. Less than 60% of non-hospitalized persons experienced post-acute manifestations after three months post-diagnosis, whereas for hospitalized patients the odds of post-acute symptoms were elevated 10% or more. Over 70% of the patients who required mechanical ventilation had post-acute symptoms after three months. Others have reported that illness severity relates to post-acute symptoms [6,19–22]. We extend those observations to a large pool of persons with minimal to moderate symptoms with lower, but non-negligible likelihood of post-acute manifestations. Although their risk is lower, they contribute much more to the population burden of post-acute COVID-19 since their numbers are so much greater than hospitalized patients. We estimate that in the United States, of the 25 million persons who experienced post-acute illness during the first 16 months of the pandemic, 90% were not hospitalized.

The overall symptoms observed at the four-week mark tended to improve gradually by three and six months post-initial COVID-19 episode, although appreciable levels of symptoms persisted, and these remained correlated with initial disease severity. The same general pattern of overall post-acute symptoms was observed for both influenza and pneumonia, with a strong gradient of risk with increasing severity of initial illness, a slow and partial resolution of symptoms over time, and the gradient of risk with disease severity persisting even at 6 months post-diagnosis. The parallel patterns here suggest that, at least to some extent, the post-acute symptoms observed with COVID-19 follow a general tendency for serious respiratory illnesses to produce persistent symptoms beyond the acute phase. The actual post-acute symptoms experienced tended to differ somewhat for the three respiratory infections examined, and likely reflect differences in the age distributions of the persons affected, the corresponding levels of comorbidities, and the pathophysiology of each illness.

The prolonged use of mechanical ventilation corresponds with extended stays in intensive care units. A study conducted at Dutch hospitals of patients who survived 1 year following ICU treatment for COVID-19 found that physical, mental, or cognitive symptoms were frequently reported, suggesting a possible similarity to post intensive care syndrome [23]. Post intensive care syndrome (PICS) refers to ongoing disability following survival from a critical illness that required treatment in an intensive care unit. Typical components seen in PICS include impairments in cognition, psychological health, neuromuscular weakness, and physical function [24]. Use of prolonged mechanical ventilation has been strongly associated with PICS. Specifically, ventilator-induced lung injury has been shown to occur most readily in patients with concomitant physiological insults to the immune system [25]. As noted earlier, such treatment has been strongly associated with post-intensive-care-syndrome, and ventilator-induced lung injury in particular. In the present study it was noted that 11.1% of the COVID-19 patients, 5.8% of the Influenza patients and 31.4% of the pneumonia patients received mechanical ventilation. While PICS may account for part of the excess risk of post-acute symptoms in mechanically ventilated patients, for all three respiratory infections considered here, half or more of all patients hospitalized without requiring oxygen therapy still had post-acute symptoms at six months.

There are several limitations of this study. First, it was based upon administrative data, which may lack the completeness and accuracy of information gathered for research purposes [26]. Follow-up times were not scheduled at pre-specified intervals, as would be typical in a research protocol, and the extent to which post-acute symptoms were fully reported and recorded is unknown. Although follow-up intervals varied, the overall median duration of follow-up was nearly five months, with a median of four evaluation episodes. There was ample opportunity, therefore, to characterize the status of each subject one month after diagnosis and beyond. Since the percentages of hospitalized subjects with post-acute manifestations paralleled prior studies, it seems unlikely that there was under- or over-reporting of post-acute symptoms.

Confounding could be present by either inadequately controlled characteristics or other unknown determinants of post-acute illness. It has been demonstrated elsewhere [Arnold] that adjusting for covariables in administrative data does not necessarily produce the same results as obtained in clinical data. There is at least the possibility, therefore, that residual confounding was present here. The association between COVID-19 severity and post-acute symptoms was so strong and consistent with the results of prior clinical studies, that confounding is an unlikely explanation for the entire observed association. Since the age distributions were different between the three cohorts, it would be wise for future research to perform age group specific analyses to determine if these trends persist when looking at a more homogenously aged population. Since our study focused on the early stages of the COVID-19 pandemic, well-established or validated treatments, like antivirals, were not yet used to treat COVID-19 like they could have been for influenza or pneumonia cases. It is possible that these treatments affected the lifespan of influenza and pneumonia symptoms such that the differences between cohorts were skewed.

In addition, the database included only persons with commercial health insurance or who participated in the Medicare Advantage private insurance option. This provides reassurance that cost of care was not a barrier to medical care and follow-up. Nevertheless, these exclusions limit the generalizability to persons who lacked commercial health insurance. For example, it is possible that the patterns seen across racial and ethnic groups in these cohorts are not reflective of the total population of COVID-19 patients, including the uninsured, those on Medicaid, and those in fee-for-service Medicare.

Despite limitations of administrative data, it is readily available and provides an efficient means to explore questions such as risk of post-acute COVID-19. Future studies should build upon the present findings by exploring how time-to-resolution of symptoms varies by prior health status, severity of illness, and other factors. Also, it will be important to determine whether prior COVID-19 vaccination, or COVID-19 treatments such as monoclonal antibodies and steroids, can reduce post-acute risk. In the meantime, the present results indicate that post-acute COVID-19 continues to be a significant burden for many patients following COVID-19 infection and for the health care system trying to manage these patients.

We examined post-acute COVID-19 risk across severity levels of symptomatic COVID-19 disease among adults in a large U.S. national administrative claims dataset. We found that COVID-19 symptomology could not be perfectly described by previously seen influenza or pneumonia symptomology. We found differences in the prevalence of symptoms as well as different times to resolution better characterizing “long COVID” and identifying that this persistence of symptoms is unique to COVID-19.
